# Early Recognition of Neuroleptic Malignant Syndrome Without Hyperthermia: A Narrative Review

**DOI:** 10.7759/cureus.114485

**Published:** 2026-08-13

**Authors:** Temebi W Andafa, Sheriffdeen A Adekanmbi, Chijioke M Ugwumba, Woyengidoubara T Angaye, Godbless N Oyinke, Zulikhat Maruwf

**Affiliations:** 1 Psychiatry, Greater Manchester Mental Health Trust, Manchester, GBR; 2 Psychiatry, Thornford Park Hospital, Elysium Healthcare, Thatcham, GBR; 3 Emergency Medicine, St. Mary's Hospital, Newport, GBR; 4 Internal Medicine/Cardiology, Niger Delta University Teaching Hospital, Yenagoa, NGA; 5 Community Medicine, Bayelsa Medical University, Yenagoa, NGA; 6 Healthcare Financing, Niger Delta University, Yenagoa, NGA; 7 Family and Community Medicine, Community Health Alliance, Reno, USA

**Keywords:** afebrile presentation, creatine kinase, diagnostic criteria, dopamine antagonists, early diagnosis, early recognition, hyperthermia, muscle rigidity, neuroleptic malignant syndrome

## Abstract

Neuroleptic malignant syndrome (NMS) is a rare but potentially fatal idiosyncratic reaction to dopamine antagonism, traditionally recognised by hyperthermia, muscular rigidity, autonomic instability, and altered mental status. International consensus and DSM-5 research criteria uniformly require documented repeated hyperthermia to establish a diagnosis. Primary cohort, case-register, pharmacovigilance, and clinical-feature-frequency data document that a clinically important minority of diagnosed patients are afebrile at presentation, and that rigidity precedes measured hyperthermia in the majority who eventually become febrile. This review synthesises the evidence bearing on early recognition of NMS in patients who do not yet meet the fever criterion, and asks what earlier markers, clinical patterns, and management steps are supported by primary evidence. Feature-frequency series show that rigidity and rising creatine kinase (CK) are earlier and more universal markers than measured hyperthermia; pathophysiological modelling is consistent with the view that hyperthermia is a downstream and variable manifestation of the underlying pathophysiological process; and outcome data indicate that delayed recognition tracks with the leading causes of death and disability (respiratory failure, acute kidney injury, rhabdomyolysis). Because all available evidence is retrospective, cohort, pharmacovigilance, or observational in design, conclusions are directional rather than definitive. Awareness of the afebrile presentation, structured attention to rigidity and CK trajectory, and early exclusion of alternative diagnoses are supported by the primary literature as low-cost strategies to shorten the time to recognition. Formal criteria are unchanged; the change proposed here is one of clinical awareness at the bedside.

## Introduction and background

Neuroleptic malignant syndrome (NMS) is an uncommon but potentially fatal reaction to dopamine antagonism, precipitated most often by antipsychotic drugs and less often by antiemetics, by abrupt withdrawal of dopamine agonists in Parkinson's disease, and by other dopaminergic agents. Its cardinal features are typically described as hyperthermia, muscular rigidity, altered mental status, and autonomic instability, together with elevated creatine kinase (CK) and leucocytosis. Every widely used current framework, including the international expert consensus (IEC) criteria developed by Delphi method in 2011 and the Diagnostic and Statistical Manual of Mental Disorders, Fifth Edition (DSM-5) research criteria, requires measured hyperthermia (>38.0°C on two or more occasions) for diagnosis [1,2].

Primary cohort, case-register, and pharmacovigilance studies document that a proportion of clinically diagnosed patients present with attenuated, delayed, or absent hyperthermia while other cardinal features are present [3-5]. This matters clinically because patients who do not yet meet the fever criterion are often not classified as suspected NMS, and their treatment is defaulted to work-ups for sepsis, serotonin syndrome, malignant catatonia, or "altered mental status of uncertain cause"; during that period, dopamine antagonists may be continued or reintroduced and the leading complications of NMS (respiratory failure, acute kidney injury, rhabdomyolysis) can accumulate.

This review asks a clinical rather than a nosological question: what does the primary evidence tell clinicians about whether earlier markers, specifically muscular rigidity, rising CK, altered mental status, and autonomic instability, can facilitate earlier recognition of NMS before hyperthermia develops, and what management steps are supported by the available evidence? The pathophysiological literature has moved toward sympathoadrenal and thermoregulatory models in which hyperthermia is a downstream manifestation rather than a defining pathological feature [6-8]. Feature-frequency, temporal-course, and criteria-validation data reviewed below document that rigidity precedes measured hyperthermia in the majority of patients who become febrile and that even the most rigorously developed criteria fail to capture a meaningful fraction of expert-adjudicated cases. The review draws these strands together around the practical question of early recognition.

Three limitations shape any conclusion. No randomised or prospective controlled study bears directly on the diagnostic value of individual features in early NMS; primary evidence is retrospective, observational, cohort, and pharmacovigilance in design. Different diagnostic criteria applied to the same patients produce order-of-magnitude differences in apparent incidence, so no criteria set can be treated as a gold standard [9,10]. Feature-frequency estimates are drawn from heterogeneous cohorts and are subject to selection and ascertainment bias.

## Review

Methods

This narrative review was informed by structured searches of PubMed/MEDLINE, Scopus, and Semantic Scholar, supplemented by citation chaining, and included English-language literature published up to July 2026. Search terms combined "neuroleptic malignant syndrome" with terms for early recognition, clinical feature frequency, atypical presentation, diagnostic criteria, thermoregulation, cohort and case-register data, and pharmacovigilance databases. Primary human studies directly addressing the review question were prioritised in the following order of evidential weight: prospective and retrospective cohort studies; population-based and register-linkage studies; case-register analyses of electronic medical records; case-control studies; multi-country pharmacovigilance database analyses; clinical-feature-frequency series in defined populations; and criteria-validation studies. Individual case reports are cited only for historical context and are not used to support the principal conclusions. No pooled statistical analysis or meta-analysis was performed because the included studies differ substantially in design, ascertainment, diagnostic criteria, and reporting granularity.

Epidemiology and outcomes: why early recognition matters

Reported incidence of NMS varies more than an order of magnitude across studies, driven predominantly by study design and diagnostic-criteria stringency. Prospective cohorts using pre-specified operational criteria produce estimates around 0.9% among neuroleptic-exposed inpatients [11]. Modern denominator-based studies produce lower estimates: a Hong Kong population-based cohort of 297,647 antipsychotic-exposed patients reported an incidence of 0.11% [12]; a Finnish nationwide case-control study of 172 cases reported 1.99 per 10,000 person-years, with no significant difference in risk by antipsychotic formulation or class [13]; and a US Medicaid cohort reported 8.9 cases per 100,000 person-years, with substantially higher incidence for first-generation antipsychotics (FGAs) than second-generation antipsychotics (SGAs) (HR 4.32, 95% CI 2.74-6.82), in contrast to the Finnish cohort [14]. Mortality in modern series is typically reported at 4-6%; the leading complications and predictors of death are acute respiratory failure, acute kidney injury, and rhabdomyolysis, not hyperthermia itself [13,15]. Because these complications are the mechanisms by which patients deteriorate, and each is a target of straightforward supportive management, the practical value of early recognition is defined by how much earlier the diagnostic suspicion can be raised, not by how strict or permissive the eventual formal diagnosis proves to be.

Why current diagnostic criteria may impede early recognition

Because the practical question is when suspicion should be raised, a structured look at how the principal criteria sets treat measured temperature is warranted. Table 1 summarises the main criteria sets in current use or in the recent research literature.

**Table 1:** Principal diagnostic criteria for neuroleptic malignant syndrome and the role of hyperthermia AUC: area under the curve; BP: blood pressure; CK: creatine kinase; DSM: Diagnostic and Statistical Manual of Mental Disorders; IEC: international expert consensus; NMS: neuroleptic malignant syndrome; ULN: upper limit of normal.

Diagnostic criteria/ Ref.	Hyperthermia status	Rigidity status	Other required features	Evidence base/validation
Gurrera IEC (2011) [1]	Required (>38.0°C on ≥2 occasions)	Required	Recent dopamine antagonist exposure, altered mental status, CK ≥4× ULN, autonomic instability, tachycardia + tachypnoea, negative work-up for other causes	Validated against expert clinician judgement: sensitivity 69.6%, specificity 90.7%, AUC 0.857
DSM-5 (2013) [2]	Required (temperature elevation on ≥2 occasions)	Required (generalised rigidity)	At least two of: diaphoresis, dysphagia, tremor, incontinence, altered consciousness, mutism, tachycardia/labile BP, leucocytosis, elevated CK	Retains hyperthermia as a required criterion; independent validation data remain limited
Levenson (1985) [16]	Required (major criterion)	Required (major criterion)	Elevated CK, leucocytosis, autonomic dysfunction (2 of 5 minor)	Widely used historically; broad sensitivity but low inter-rater agreement in real-world case registers
Pope et al. (1986) [17]	Required (>37.5°C)	Required (extrapyramidal)	Autonomic dysfunction + one additional feature	Yielded an approximately 10-fold higher apparent incidence than Adityanjee's criteria when applied to the same cohort
Adityanjee et al. (1999) [18]	Required	Required (severe)	Autonomic dysfunction, altered consciousness, CK, exclusion of other causes	Proposed research diagnostic criteria intended to improve diagnostic specificity
Caroff and Mann (1993) [19]	Required	Required (severe)	Two of six additional (mental status, autonomic instability, CK, leucocytosis, tremor, incontinence)	Influential framework underpinning later DSM diagnostic criteria
DSM-IV / DSM-IV-TR (1994/2000) [20]	Required (severe; typically >39.0°C)	Required (severe)	Two of 10 additional features	Consultant diagnoses agreed better with a modified DSM-IV-TR standard accepting less than "severe" rigidity than with the original

Every widely used set requires documented hyperthermia; the observed sensitivity-specificity trade-off appears to be influenced substantially by criteria design rather than biological variation alone. Tsutsumi and colleagues quantified this directly within a single 888-inpatient cohort by applying both Pope's and Adityanjee’s criteria: apparent incidence was 2.36% under Pope and 0.23% under the stricter set, a 10-fold difference from the same underlying patients driven only by criteria choice [10]. Chang and colleagues extended the finding to six criteria sets applied to 183 suspected NMS cases in the South London and Maudsley electronic medical records register: only 43 (23.5%) met at least one set, only one met all six, and agreement between criteria was poor (κ = 0.35, 95% CI 0.31-0.39) [8]. The IEC criteria, the most rigorously developed set available, showed a validated sensitivity of 69.6% and specificity of 90.7% against a modified DSM-IV-TR reference standard [20,21]. Sensitivity below 70% against expert clinician judgement suggests that approximately three in 10 clinically identified cases may fall outside the formal definition, indicating limitations of the current criteria rather than shortcomings in expert clinical judgement. For early recognition, the implication is direct: a formal diagnosis of NMS is a lagging indicator, not a bedside trigger. Clinicians who wait for two documented temperatures above 38.0°C before considering the diagnosis will identify NMS at the point at which the fever criterion has already been met, not at the point at which the earliest features first appeared.

Which features come first? Evidence for earlier markers than hyperthermia

The most direct evidence addressing early recognition comes from cohort and clinical-feature studies of NMS that examined the frequency and temporal sequence of its cardinal clinical features. Four studies are particularly informative. Velamoor et al. (n = 212) evaluated symptom frequencies and their relationship with temperature [3]; Amrat et al. (n = 55) described the clinical and laboratory features present at diagnosis [4]; Trollor et al. (n = 208) compared the clinical characteristics of NMS associated with first- and second-generation antipsychotics [5]; and Nisijima et al. (n = 24) examined the temporal relationship between muscular rigidity, fever, and CK during the course of the illness [22]. These studies describe the distribution of temperature within NMS, the proportion of patients who are afebrile at presentation, the frequency of the remaining cardinal features, and the sequence in which they develop. Their findings complement one another and provide stronger support for the conclusions of this review than any individual study alone.

Distribution of temperature within NMS cohorts

The temperature range recorded across large cohort data is much wider than a strict diagnostic threshold suggests. Velamoor's 212-case FGA-treated series recorded temperatures ranging from 37.2°C to 43.0°C, with a mean of 39.5°C (standard deviation [SD] 1.3) [3], and Amrat's Karachi cohort confirmed that a substantial proportion of patients presented below the 38.0°C threshold [4]. Trollor's pharmacovigilance comparison of 208 FGA- and SGA-treated cases found similar frequencies of fever across antipsychotic generations, suggesting that this broad temperature distribution is not specific to a particular drug class but is a feature of the syndrome itself [5]. Interpretation is limited by the archival nature of Velamoor's dataset, the single-centre design of Amrat's study, and the reporting biases inherent in pharmacovigilance data. Nevertheless, the consistency of these findings across different study designs supports the conclusion that the temperature spectrum in NMS is broader than a strict fever threshold implies.

Proportion of afebrile presentations

Amrat's prospective cohort reported fever in 81.8% of patients at presentation, giving an afebrile proportion of approximately 18% [4]. Velamoor's data implicitly triangulate this figure: with a minimum recorded temperature of 37.2°C, some proportion of the 212-patient FGA-treated series fell at or below the 38.0°C IEC threshold [3]. Because both cohorts were identified using diagnostic criteria that required or favoured hyperthermia, the true prevalence of afebrile presentation is likely to be underestimated. Nevertheless, the consistency of findings across historical and contemporary cohorts suggests that afebrile presentation is a genuine feature of NMS rather than one confined to a particular era or drug class.

Frequency of other cardinal features and their independence from hyperthermia

Rigidity is the most consistently reported feature: Velamoor recorded it in 91.0% [3] and Amrat in 69.1% of patients [4], with Trollor documenting significantly lower rigidity only in clozapine-induced cases within the SGA subset [5]. Autonomic instability (66.5% in Velamoor [3], 38.2% in Amrat [4]) and altered mental status (Amrat 56.4% [4]; consistently reported in Velamoor's mutism and stupor subcategories [3]) are common but less universal. CK elevation was present in 47.3% of Amrat's cohort [4] and is not always reported in the archival Velamoor set. Crucially, Velamoor's within-cohort correlation analysis found that higher temperature correlated significantly only with tachycardia and with severe impairment of consciousness or coma, not with rigidity, autonomic instability, diaphoresis, or the other cardinal features [3]. Temperature therefore tracks the acuity end of the clinical spectrum rather than the syndrome's core features. For early recognition, rigidity, not hyperthermia, is the most sensitive and most consistently present feature.

Temporal sequence and CK kinetics

Nisijima's 24-patient temporal cohort reported that mild muscle rigidity preceded fever onset in 71% of cases, that CK peaked on day two after fever onset, and that rigidity progressed until day four [22]. Because that sample was drawn from patients who did ultimately develop fever, it does not directly address afebrile cases, but read against Amrat's cross-sectional 18% afebrile-at-presentation figure [4], the combined temporal picture is that rigidity and CK rise are earlier markers than fever in nearly three-quarters of patients who become febrile, and that a clinically important minority do not cross the fever threshold during clinical presentation. A criterion that requires two temperature measurements above 38.0°C therefore builds a systematic delay into recognition even in the majority who eventually meet it. Limitations are the small sample and the exclusion of never-febrile cases, both of which mean the finding should be interpreted directionally rather than quantitatively.

Findings across drug generations and clinical contexts.

Trollor's Australian Adverse Drug Reaction Advisory Committee (ADRAC)-based comparison is the strongest available primary evidence on generational effects: features were largely similar across FGA and SGA groups, with lower rigidity rates specifically in clozapine cases, and mortality substantially lower in the SGA group (3.0% vs 16.3%) [5]. Velamoor's FGA-only cohort and Amrat's mixed-agent cohort both report atypical (afebrile or low-temperature) presentations, so the phenomenon is not an SGA-era artefact and cannot be attributed to a class-specific pharmacology. The lower mortality reported in the SGA cohort in Trollor's data more plausibly reflects improvements in recognition and supportive care than a change in the underlying syndrome, and it is consistent with the pattern reported in modern denominator-based cohorts [13,15]. Clinicians should not assume that SGA-treated patients present differently or later than FGA-treated patients; the same afebrile phenotype occurs in both.

Across these four studies, no single finding is beyond challenge, but the direction of evidence is consistent: rigidity is a more frequent, earlier, and more universal marker than fever; approximately one in five patients is afebrile at presentation; higher temperature within diagnosed cohorts tracks acuity rather than diagnosis; and generational differences affect specific features rather than the fever criterion globally. All four studies are retrospective or single-centre, and each was ascertained using diagnostic criteria that themselves required or favoured hyperthermia; the observed proportion of afebrile NMS should therefore be treated as a minimum estimate. Table 2 summarises the numerical detail.

**Table 2:** Primary cohort and clinical-feature series bearing on the frequency and timing of hyperthermia in NMS ADRAC: Australian Adverse Drug Reaction Advisory Committee; CK: creatine kinase; FGA: first-generation antipsychotic; GCS: Glasgow Coma Scale; NMS: neuroleptic malignant syndrome; SD: standard deviation; SGA: second-generation antipsychotic; WBC: white blood cell count.

Study, year / Ref.	Design	Population	Key finding on temperature	Other principal findings	Main limitation
Velamoor et al., 2021 [3]	Archival multi-source case series	212 suspected NMS cases (FGA-treated)	Temperature 37.2–43.0°C; mean 39.5°C (SD 1.3); correlated significantly only with tachycardia and severe consciousness impairment	Rigidity 91.0%; autonomic instability 66.5%; diaphoresis 45.8%; mutism 34.4%; tremor 31.6%	Archival data; selection bias in submissions
Amrat et al., 2022 [4]	Prospective observational	55 NMS patients, single tertiary neurology centre	Fever present in 81.8% (~18% afebrile at presentation)	Extrapyramidal signs 69.1%; altered GCS 56.4%; elevated CK 47.3%; elevated WBC 74.5%	Small sample; single centre
Trollor et al., 2012 [5]	Retrospective pharmacovigilance database analysis	208 NMS reports (FGA vs SGA), ADRAC	Fever rates similar across generations	Rigidity lower in SGA cases (driven by clozapine); mortality 3.0% (SGA) vs 16.3% (FGA)	Pharmacovigilance database (under-reporting, stimulated reporting)
Nisijima et al., 2013 [22]	Retrospective cohort of temporal course	24 NMS patients (all developed fever)	Rigidity preceded fever onset in 17/24 (71%)	CK peaked day 2 post-fever; returned to normal by day 12; rigidity worsened until day 4	Small sample; retrospective; excludes never-febrile cases

Pathophysiology: why hyperthermia is a late and variable output

The mechanistic literature contains three principal models. Despite differences in emphasis, the three models are broadly consistent regarding the role of hyperthermia in NMS, and the convergence supports the same clinical implication as the feature-frequency data.

All three regard hyperthermia as a downstream and variable manifestation of the underlying pathophysiological process rather than its defining pathological feature. Gurrera's sympathoadrenal model holds that loss of tonic inhibitory dopaminergic input to preganglionic sympathetic neurons releases the sympathetic nervous system's latent capacity for autonomous activity, producing muscle tone, thermogenesis, and multi-organ dysfunction; hyperthermia is one output of this dysregulation but not the primary event [6]. Gurrera's thermoregulatory study of 36 patients across 46 NMS episodes found that thermoeffector responses did not organise into either a hypothalamic set-point elevation (as in infection) or a peripheral heat-load response (as in classical heat stroke); thermoeffector activity appeared to function paradoxically and independently, suggesting a loss of integrated thermoregulatory control [7]. Gillman's Hill-criteria analysis reached the same downstream framing from a different direction: temperatures above 39.5°C cause physiological and cellular dysfunction, and irreversible changes occur around 40°C, but most reported NMS episodes involve temperatures below 39°C, which sits within the normal mammalian stress range [8].

The three models differ on how strongly the causal link between antipsychotic exposure and hyperthermia should be characterised. Gurrera's models have been described by their author as hypotheses rather than established mechanisms and rely primarily on physiological reasoning and observational human data [6]. Gillman's application of the Hill criteria produced a stronger conclusion: that the causal case for antipsychotics as an efficient cause of hyperthermia in NMS is weaker under Hill's criteria than the equivalent case for malignant hyperthermia or serotonin toxicity, and that the label "hyperthermia" is applied loosely because most NMS temperatures do not reach the cellular-damage range [8]. Gillman's position is a minority view within the field and has not been operationalised into criteria; interpretation is limited by the inferential nature of the Hill-criteria approach and by the observation that most primary sources on which Gillman relied were themselves retrospective. The underlying empirical observation is nonetheless consistent with the feature-frequency data reviewed above [3,4].

Taken together, these mechanistic models support the biological plausibility that hyperthermia represents a variable downstream manifestation of NMS rather than its defining pathological event. For early recognition, the mechanistic evidence points in the same direction as the feature-frequency evidence: the earliest expressions of the pathogenic process are dopaminergic disinhibition, sympathetic dysregulation, and rigidity; measured hyperthermia is a variable output of these, depending on ambient conditions, thermoregulatory reserve, and timing. Clinicians who monitor for the earlier expressions can raise suspicion before the downstream output declares itself.

The counter-argument: specificity, diagnostic drift, and risk of overdiagnosis

Any argument for earlier recognition must engage with the historical experience that permissive suspicion produced diagnostic drift. Adityanjee and colleagues argued that higher incidence figures in earlier retrospective studies were driven by loose criteria and by adherence to an amorphous "spectrum concept," and that stringent prospective studies produced lower and more consistent estimates [23]. Gurrera's meta-analytic examination of 26 incidence estimates found that study size accounted for 90.8% of the variance across studies (β = 0.953, P < 0.001), supporting the view that permissive criteria yielded inflated denominators [24]. Hasan and Buckley's review of 32 clozapine and risperidone case reports designated fewer than half as high-probability NMS after exclusionary work-up, cautioning that side-effect profiles of atypical agents overlap considerably with NMS criteria and that neurotoxicities may be misclassified as NMS [25]. The meta-analytic evidence is limited by heterogeneity of underlying study designs and by the fact that criteria stringency was itself a proxy for study quality. The case-review evidence is limited by the small number of cases in each set and by the retrospective nature of adjudication.

On this view, hyperthermia contributes disproportionately to specificity: rigidity, altered mental status, autonomic instability, and CK elevation each occur in many other syndromes, but their combination with measured fever in a patient on a dopamine antagonist is highly specific for NMS. The IEC priority-point weighting is offered as a route to atypical presentations without redefining the anchors [21]. This is not a counter-argument against early recognition; it is a counter-argument against dropping fever from the diagnostic criteria. Early recognition and criteria stringency are separable goals: a clinician can raise suspicion, discontinue the offending drug, and start supportive care in a patient who does not yet meet the fever criterion without ever formally coding that patient as NMS if the fever criterion is not ultimately met.

Integration and discussion

Three complementary lines of evidence reviewed above (feature-frequency within NMS cohorts, diagnostic-criteria validation and register agreement, and pathophysiological modelling) converge on a specific answer to the review question, but each is limited in ways that constrain how far the answer can be pushed. Taken together, the available evidence supports a temporal model of NMS in which dopamine blockade produces early neuromuscular and autonomic dysfunction, followed by biochemical injury and only later, in many patients, clinically significant hyperthermia. Each of the sections above can be read as evidence bearing on this temporal model.

Feature-frequency data show that rigidity is a more frequent, earlier, and more universal marker than hyperthermia within diagnosed cohorts: rigidity is present in 91% of patients and precedes fever in 71% of those who become febrile, whereas a clinically important minority of diagnosed patients are afebrile at presentation. Criteria-validation evidence shows that even the most rigorously developed criteria are sensitive to only about 70% of expert-adjudicated cases, agreement across published criteria in real case registers is poor, and criteria choice alone can produce order-of-magnitude differences in apparent incidence. Pathophysiological modelling is consistent with hyperthermia as a downstream and variable manifestation of the underlying pathophysiological process, though the models are hypotheses derived from inferential and observational data.

Against this background, the clinical implication is that early recognition should not depend on documented fever. Waiting for the fever criterion in a patient who otherwise meets the remaining IEC criteria may delay recognition and treatment, increasing the risk of complications such as rhabdomyolysis, acute kidney injury, and respiratory failure [15]. Because rigidity precedes fever in the majority of patients who develop it and a clinically important minority never cross the threshold at all, an approach that treats rigidity, altered mental status, autonomic instability, and rising CK as triggers for clinical suspicion, followed by discontinuation of the offending agent, structured monitoring, and early exclusion of alternative diagnoses, is supported by primary evidence as a low-cost strategy to shorten the time to action.

The strength of the evidence differs across recommendations. The case for rigidity as an early marker derives from multiple independent cohort and temporal studies with consistent findings, so confidence there is moderate. By contrast, evidence for pharmacological adjuncts is limited to small observational studies and one prospective comparison, so confidence there is low. Consequently, confidence is substantially greater for recommendations concerning early recognition than for recommendations concerning adjunctive treatment. Despite NMS being recognised for over five decades, no prospective diagnostic study has evaluated patients before fever develops; the entire evidence base for the early-recognition question is derived from patients ascertained after criteria were met. Two research questions follow directly. The first is a prospective cohort study of clinically suspected NMS that records temperature trajectories at fixed intervals and compares them to the full IEC anchor set, permitting direct estimation of the proportion of clinically managed cases that fail the fever criterion. The second is a case-control comparison of outcomes between patients treated for NMS on the basis of the remaining criteria without documented fever and those in whom treatment awaited fever, though ethical constraints on such a design are considerable.

Clinical implications: recognising NMS before hyperthermia is documented

The primary evidence supports a practical bedside approach centred on three questions: who to suspect, what to monitor, and what to do when suspicion is raised.

Any patient receiving a dopamine antagonist (antipsychotic, antiemetic, or other dopaminergic agent), or with recent withdrawal of a dopamine agonist, who develops new-onset rigidity, altered mental status, or autonomic instability should be considered a possible case of NMS, regardless of measured temperature. Suspicion should be raised particularly by (a) new or progressive muscular rigidity, given that it precedes fever in the majority of patients who subsequently become febrile [22]; (b) unexplained altered mental status or mutism in a patient exposed to a dopamine antagonist [3,4]; (c) diaphoresis, tachycardia, or blood pressure fluctuation without an obvious infectious focus [3,5]; and (d) a rising CK without an obvious muscular or traumatic cause [4,22]. Because generational differences do not appear to affect the fever criterion overall [5], the same level of suspicion should be maintained for patients receiving FGAs and SGAs. Clozapine-induced NMS may present with less prominent rigidity [5], so the absence of rigidity in a patient receiving clozapine should not be considered reassuring in isolation.

In patients with suspected NMS who do not yet meet the full IEC criteria, structured monitoring should include repeated core temperature measurements to detect fever if it develops, serial CK measurements to identify the characteristic rise that typically peaks around day two following fever onset [22], repeated assessment of rigidity to recognise the mild and evolving forms described in Nisijima's temporal cohort [22], monitoring of consciousness (Glasgow Coma Scale or equivalent) and autonomic signs, and surveillance for the major complications of NMS, including rhabdomyolysis, renal impairment, respiratory compromise, and reduced urine output. These measures are inexpensive, readily available, and feasible in general ward and emergency department settings.

Withdrawal of the offending dopamine antagonist (or reinstitution of the withdrawn dopamine agonist) is the only intervention consistently supported across the primary evidence and should not be deferred pending confirmation of the fever criterion. Aggressive supportive care with attention to hydration, renal protection, and thermoregulation remains the cornerstone of management. Pharmacological adjuncts such as bromocriptine, dantrolene, benzodiazepines, and electroconvulsive therapy may be used selectively, but the primary evidence supporting these interventions remains limited. Indeed, the only prospective comparative study found that dantrolene and bromocriptine, either alone or in combination, tended to prolong illness compared with supportive care alone [26]. Alternative diagnoses, including sepsis, serotonin syndrome, malignant catatonia, encephalitis, and thyroid storm, should be investigated in parallel rather than sequentially, as their overlap with early NMS frequently contributes to delayed recognition. Reintroduction of a dopamine antagonist in a patient with unresolved suspicion should be avoided until an alternative diagnosis has been convincingly established.

This review is intended to raise clinical awareness that a meaningful proportion of patients with NMS will not meet the fever criterion at the time when withdrawal of the offending agent and supportive care would be most beneficial. The earliest and most sensitive markers, namely rigidity, altered mental status, a rising CK, and autonomic instability in a patient receiving a dopamine antagonist, are available at the bedside well before hyperthermia is documented and are accessible to the clinician who is looking for them.

Limitations

Several limitations bear on the strength of the review's conclusions. No randomised or prospective controlled evidence bears directly on the value of individual features for early recognition. Cohort and case-register studies rely on the diagnostic criteria whose adequacy is under discussion, so their check on the argument is only partial and the true proportion of afebrile NMS is likely underestimated. The four principal feature-frequency series differ in ascertainment, population, and sample size and cannot be pooled without loss of information. The pathophysiological models are hypotheses derived from inferential and observational data. Evidence for pharmacological adjuncts is limited to small case series and open-label reports; the single prospective comparison identified no adjunct benefit for dantrolene or bromocriptine over supportive care [26]. Much of the available evidence originates from retrospective cohorts and pharmacovigilance studies, which limits causal inference and may underestimate atypical presentations because many historical diagnostic criteria required hyperthermia for case ascertainment. The review is English-language and may under-sample non-English primary studies. No formal risk-of-bias assessment was performed. Conclusions should therefore be treated as provisional and directional, and the practical recommendations offered above should be read as evidence-informed clinical suggestions rather than validated protocols.

## Conclusions

Three conclusions emerge from the available evidence. First, cohort and clinical-feature studies consistently identify a minority of patients diagnosed with NMS who present with attenuated, delayed, or absent hyperthermia while other cardinal features are present, and the phenomenon occurs across both drug generations. Second, the principal pathophysiological models are consistent with the view that hyperthermia is a downstream and variable manifestation of the underlying pathophysiological process rather than a defining pathological feature. Third, rigidity, altered mental status, autonomic instability, and rising CK in a patient on a dopamine antagonist are earlier and more sensitive markers than measured hyperthermia. The practical implication is that early recognition of NMS should not depend on documented fever: awareness of the afebrile presentation, structured monitoring of the earlier markers, prompt discontinuation of the offending agent, and parallel work-up of alternative diagnoses are supported by the primary literature as low-cost strategies to shorten the time to action. Existing formal diagnostic criteria remain appropriate for research and audit; the change proposed here is at the bedside, not in the criteria. Clinicians should regard hyperthermia as a confirmatory manifestation of NMS rather than the earliest opportunity for suspicion.

## References

[1] Gurrera RJ, Caroff SN, Cohen A (2011). An international consensus study of neuroleptic malignant syndrome diagnostic criteria using the Delphi method. J Clin Psychiatry.

[2] (2013). Diagnostic and Statistical Manual of Mental Disorders (DSM-5).

[3] Velamoor V, Mann SC, Woodbury MA, Cernovsky ZZ (2021). Symptom frequencies and correlations with temperature variations in suspected cases of neuroleptic malignant syndrome (NMS). Eur J Med Health Sci.

[4] Amrat S, Bullo N, Kumar D (2022). Frequency of pattern of clinical and laboratory features in patients presenting with neuroleptic malignant syndrome. Ro J Neurol.

[5] Trollor JN, Chen X, Chitty K, Sachdev PS (2012). Comparison of neuroleptic malignant syndrome induced by first- and second-generation antipsychotics. Br J Psychiatry.

[6] Gurrera RJ (1999). Sympathoadrenal hyperactivity and the etiology of neuroleptic malignant syndrome. Am J Psychiatry.

[7] Gurrera RJ, Chang SS (1996). Thermoregulatory dysfunction in neuroleptic malignant syndrome. Biol Psychiatry.

[8] Gillman PK (2010). Neuroleptic malignant syndrome: mechanisms, interactions, and causality. Mov Disord.

[9] Chang CK, Harrison S, Lee W, Taylor D, Stewart R (2012). Ascertaining instances of neuroleptic malignant syndrome in a secondary mental healthcare electronic medical records database: the SLAM BRC Case Register. Ther Adv Psychopharmacol.

[10] Tsutsumi Y, Yamamoto K, Matsuura S (1994). Incidence of "typical cases" and "incomplete cases" of neuroleptic malignant syndrome and their epidemiological study. Psychiatry Clin Neurosci.

[11] Keck PE Jr, Pope HG Jr, McElroy SL (1987). Frequency and presentation of neuroleptic malignant syndrome: a prospective study. Am J Psychiatry.

[12] Lao KS, Zhao J, Blais JE (2020). Antipsychotics and risk of neuroleptic malignant syndrome: a population-based cohort and case-crossover study. CNS Drugs.

[13] Guinart D, Taipale H, Rubio JM, Tanskanen A, Correll CU, Tiihonen J, Kane JM (2021). Risk factors, incidence, and outcomes of neuroleptic malignant syndrome on long-acting injectable vs oral antipsychotics in a nationwide schizophrenia cohort. Schizophr Bull.

[14] Ray WA, Fuchs DC, Olfson M, Stein CM, Murray KT, Daugherty J, Cooper WO (2024). Incidence of neuroleptic malignant syndrome during antipsychotic treatment in children and youth: a national cohort study. J Child Adolesc Psychopharmacol.

[15] Modi S, Dharaiya D, Schultz L, Varelas P (2016). Neuroleptic malignant syndrome: complications, outcomes, and mortality. Neurocrit Care.

[16] Levenson JL (1985). Neuroleptic malignant syndrome. Am J Psychiatry.

[17] Pope HG Jr, Keck PE Jr, McElroy SL (1986). Frequency and presentation of neuroleptic malignant syndrome in a large psychiatric hospital. Am J Psychiatry.

[18] Mathews T, Aderibigbe YA (1999). Proposed research diagnostic criteria for neuroleptic malignant syndrome. Int J Neuropsychopharmacol.

[19] Caroff SN, Mann SC (1993). Neuroleptic malignant syndrome. Med Clin North Am.

[20] (2000). Diagnostic and Statistical Manual of Mental Disorders text revision (DSM-IV-TR).

[21] Gurrera RJ, Mortillaro G, Velamoor V, Caroff SN (2017). A validation study of the international consensus diagnostic criteria for neuroleptic malignant syndrome. J Clin Psychopharmacol.

[22] Nisijima K, Shioda K (2013). Temporal changes in serum creatine kinase concentration and degree of muscle rigidity in 24 patients with neuroleptic malignant syndrome. Neuropsychiatr Dis Treat.

[23] Adityanjee Adityanjee, Aderibigbe YA, Mathews T (1999). Epidemiology of neuroleptic malignant syndrome. Clin Neuropharmacol.

[24] Gurrera RJ, Simpson JC, Tsuang MT (2007). Meta-analytic evidence of systematic bias in estimates of neuroleptic malignant syndrome incidence. Compr Psychiatry.

[25] Hasan S, Buckley P (1998). Novel antipsychotics and the neuroleptic malignant syndrome: a review and critique. Am J Psychiatry.

[26] Rosebush PI, Stewart T, Mazurek MF (1991). The treatment of neuroleptic malignant syndrome. Are dantrolene and bromocriptine useful adjuncts to supportive care?. Br J Psychiatry.

